# The Pilot Study of Fibrin with Temporomandibular Joint Derived Synovial Stem Cells in Repairing TMJ Disc Perforation

**DOI:** 10.1155/2014/454021

**Published:** 2014-04-15

**Authors:** Yang Wu, Zhongcheng Gong, Jian Li, Qinggong Meng, Wei Fang, Xing Long

**Affiliations:** ^1^Department of Oral and Maxillofacial Surgery, The State Key Laboratory Breeding Base of Basic Science of Stomatology & Key Laboratory of Oral Biomedicine Ministry of Education, School & Hospital of Stomatology, Wuhan University, No. 237 Luo Yu Road, Wuhan, Hubei 430079, China; ^2^Oncology Department of Oral & Maxillofacial Surgery, The First Teaching Hospital of Xinjiang Medical University, Stomatology College of Xinjiang Medical University, Stomatology Research Institute of Xinjiang Province, Urumqi, Xinjiang 830054, China

## Abstract

TMJ disc related diseases are difficult to be cured due to the poor repair ability of the disc. TMJ-SDSCs were ideal cell sources for cartilage tissue engineering which have been widely used in hyaline cartilage regeneration. Fibrin gel has been demonstrated as a potential scaffold for neocartilage formation. The aim of this study was to repair the TMJ disc perforation using fibrin/chitosan hybrid scaffold combined with TMJ-SDSCs. Rat TMJ-SDSCs were cultured on hybrid scaffold or pure chitosan scaffolds. The cell seeding efficiency, distribution, proliferation, and chondrogenic differentiation capacity were investigated. To evaluate the * in vivo* repair ability of cell/scaffold construct, rat TMJ disc explants were punched with a defect to mimic TMJ disc perforation. Cell seeded scaffolds were inserted into the defect of TMJ disc explants and then were implanted subcutaneously in nude mice for 4 weeks. Results demonstrated that fibrin may improve cell seeding, proliferation, and chondrogenic induction * in vitro*. The * in vivo* experiments showed more cartilage ECM deposition in fibrin/chitosan scaffold, which suggested an enhanced reparative ability. This pilot study demonstrated that the regenerative ability of TMJ-SDSCs seeded in fibrin/chitosan scaffold could be applied for repairing TMJ disc perforation.

## 1. Introduction

Temporomandibular joint disorder (TMDs) is frequently associated with degenerative changes in severe cases [1]. The degenerative changes including TMJ disc perforation and osteoarthritis usually need surgical repair of the disc or total joint reconstruction due to its poor intrinsic healing ability [2].

The TMJ disc is characterized as fibrocartilage tissue distinct from both hyaline and meniscal cartilage in cell type and extracellular matrix (ECM) composition [3]. Previous studies on the TMJ disc engineering usually used TMJ disc cells [4–6]. However, it was found that this kind of fibrochondrocyte was prone to dedifferentiate during* in vitro* culture, leading to a decrease of ECM synthesis [7, 8].

Synovium derived mesenchymal stem cells (SDSCs) are an attractive cell source for cartilage tissue engineering. SDSCs are able to synthesis cartilage oligomeric matrix protein, link protein, and glycosaminoglycans (sGAG), which demonstrates the same properties as chondrocytes [9]. The ability of multipotential differentiation of TMJ-SDSCs has been confirmed in our previous researches [10, 11]. Although SDSCs have been used in hyaline cartilage tissue engineering, there was no research using TMJ-SDSCs to regenerate TMJ disc tissue, which was regarded as fibrocartilage. In this study, we hypothesized that TMJ-SDSCs can be used in TMJ disc tissue engineering.

Another important aspect of cartilage tissue engineering is the design of three-dimensional scaffold which may maintain the initial shape of cell/scaffold construct and promote tissue regeneration. In our previous research, macroporous sponge-like chitosan was used as scaffold in hyaline cartilage repair [12]. Fibrin gel is a Federal Drug Agency (FDA) approved biological adhesive which possesses several essential features as a scaffold for cartilage engineering, for it promotes chondrocytes proliferation and cartilaginous ECM production [13]. However, the intrinsic properties of fibrin gel such as poor mechanical strength and fast degradation make it unsuitable to be used independently.

We hypothesize that incorporating fibrin gel with sponge-like chitosan scaffold could improve the biocompatibility of scaffold. In this study, we compared the* in vitro* results of fibrocartilage tissue engineered using TMJ-SDSCs seeded in sponge-like chitosan scaffold with or without fibrin gel incorporation. In the second phase of study, we designed an* in vivo *organ culture model to mimic the perforation of TMJ disc and tested whether fibrin gel could promote TMJ disc repair in a subcutaneous nude mice model.

## 2. Materials and Methods

### 2.1. Reagents and Chemicals

Cell culture regents including high glucose Dulbecco's modified Eagle's medium (DMEM), fetal bovine serum (FBS), and phosphate-buffered saline (PBS) were purchased from HyClone (USA); recombinant human TGF-*β*3 was obtained from Propertech (USA); ITS^+premix^ (insulin, transferring, and selenium) was purchased from Gibco (USA); chitosan with a deacetylation degree of minimum 95% was purchased from Shangon (Shanghai, China); fibrin gel was from Guangzhou BioSeal Company (China).

Other reagents were obtained from Sigma (St. Louis, MO), unless otherwise specified.

### 2.2. Preparing of Fibrin/Chitosan Scaffolds

Macroporous chitosan scaffold was prepared in the manner of freeze-drying method. In brief, chitosan was dissolved in 0.1 mol/L acetic acid solution to prepare a 1% (w/v) solution. After centrifugation at 4°C for 1000 rpm × 10 min, chitosan solution was poured into a polystyrene 48-well culture plate (0.2 mL per well) and frozen at −70°C for 24 hours and then lyophilized in a freeze dryer (Christ, Germany) for 48 hours. Scaffolds were sterilized with ethylene oxide and soaked in DMEM before use.

The major components of fibrin gel were fibrinogen (50~75 mg/mL) and thrombin (400 IU). Other components included blood coagulation-factor XIII (10~70 U), potassium dihydrogen phosphate (0.68 mg/mL), and calcium chloride (40 mmol). The fibrin gel was formed by mixing fibrinogen and thrombin solution at equal volume in 15 min at 37°C according to the manufacture's protocol.

To achieve homogeneous incorporation of fibrin gel with chitosan scaffold, fibrinogen solution (with or without cells) was dropped equally onto both sides of the half-dry chitosan scaffold. After fibrinogen solution was absorbed, thrombin solution was added to form fibrin gel. In this study, the hybrid chitosan/fibrin scaffolds were used in experiment group and pure chitosan scaffolds were used as controls.

To evaluate the morphological features of two scaffolds, cell-free scaffolds were fixed, dehydrated, critical-point dried, and coated with gold for scanning electron microscopy (SEM; Quanta 200, FEI, The Netherlands) analysis.

### 2.3. TMJ-SDSCs Isolation and Expansion

TMJ-SDSCs were isolated from the TMJ synovial membrane of 4-week-old Sprague-Dawley rats. All procedures were performed with approval by the Animal Care and Use Committee, school of Stomatology, Wuhan University. After anesthesia of animals, the TMJ capsule of rat was exposed and synovial tissue lining on the posterior band of TMJ disc was harvested aseptically under stereo microscope. Synovial tissue was cut into 1 mm^3^ piece and cultured in primary cell culture medium (DMEM with 15% FBS, 100 *μ*g/mL streptomycin, and 100 U/mL penicillin) on 25 cm^2^ flasks in a humidified incubator at 5% carbon dioxide and 37°C. When the primary cells attached to the flasks, the synovial tissue was removed. After 12 days of expansion, TMJ-SDSCs of passage 3 were harvested with 0.25% trypsin and counted for future use.

### 2.4. Preparation of Cell/Scaffold Constructs and Three-Dimensional Culture

In fibrin/chitosan group, TMJ-SDSCs were pelleted by centrifugation and resuspended with 50 *μ*L of fibrinogen solution. Cell/fibrinogen suspension was gently dropped onto the top surface of half-dried chitosan scaffold of both sides and then equal volume of thrombin solution was added onto scaffold in the same way. The cell/fibrin/chitosan construct was incubated at 37°C for 15 min to polymerize the fibrinogen, and then gently the construct was washed with PBS and cultured with culture medium in 24-well plate.

In control group, pure chitosan was used as scaffold. The same amount of cells was resuspended with 100 *μ*L of culture medium. Half of the suspension was gently dropped onto the top surface of half dried scaffold. After an interval of 2.5 hours, the rest of cell suspension was dropped onto the other side of scaffold. The cell/chitosan scaffold was washed and transferred into 24-well plate with culture medium after 3 hours of cell seeding.

### 2.5. *In Vitro* Studies on Cell Seeding, Distribution, Expansion, and Chondrogenic Induction

TMJ-SDSCs seeded scaffolds (2 × 10^6^ cells per scaffold) with (experiment group) or without fibrin incorporation (control group) were cultured in 24-well plate with 2 mL culture medium per well at 37°C in a humidified 5% CO_2_/95% air incubator.

After 8 hours of cell seeding, cell/scaffold constructs (*n* = 8 of each group) were transferred to a blank well of plate, and the rest of the cells which were attached to the former well were trypsinized and counted. Cell seeding efficiency was calculated as follows: (total number of cells − rest cells)/total number of cells × 100%.

Cell vitality and distribution after 5 days of cell seeding were assessed using fluorescein diacetate/propidium iodide (FDI/PI) staining. Constructs (*n* = 4 of each group) were rinsed with PBS and incubated with 5 *μ*g/mL FDA solution for 15 min at 37°C in the dark. Then, the constructs were rinsed again and incubated within 0.1 mg/mL PI solution for 2 min at room temperature. After an additional washing step, the cell vitality and distribution of the constructs were analyzed with a Confocal Laser Scanning Microscopy (CLSM, Leica TCS SP2, Germany).

Cell proliferation ability among the two groups was measured with the Cell Counting Kit-8 (CCK-8, Dojindo Laboratories, Japan). Cell/scaffold constructs (*n* = 6 of each group) were cultured in 24-well plate with culture medium. The medium was renewed at 3-day interval. At days 1, 7, 14, 21, and 28, the culture medium was changed into incubating medium containing 100 *μ*L of CCK-8 solution and 900 *μ*L of fresh culture medium. After 4 hours of incubation at 37°C, the incubating medium was measured using a microplate absorbance reader (Varioskan Flash, Thermo Electron Corporation, USA) at a wavelength of 450 nm. Blank well with fresh culture medium was used for the zero setting.

To evaluate the influence on chondrogenic induction after fibrin incorporation, experiment and control groups of cell/scaffold constructs were cultured in 24-well plate with serum free chondrogenic medium, including high glucose DMEM, 100 *μ*g/mL ascorbic acid 2-phosphate, 100 *μ*g/mL sodium pyruvate, 100 *μ*g/mL streptomycin, 100 U/mL penicillin, 40 *μ*g/mL proline, 100 *μ*M dexamethasone, 1 × ITS^+Premix^, and 10 ng/mL TGF-*β*3. The chondrogenic medium was renewed every other day. At days 0, 14, and 28, constructs were biochemically analyzed.

*Biochemical Analysis*. sGAG and collagen are the main components of cartilaginous ECM. sGAG to DNA ratio represents the synthetic ability of cells. It was performed by incubating the constructs (*n* = 5 of each group) in 1 mL of papain buffer solution (5 mg/mL in 0.2 M NaCl, 0.05 M Na_2_-EDTA, 0.1 M NaAc, and 0.01 m L-cysteine-HCl, pH 6.0) at 60°C for 24 h. The sGAG content of each construct was evaluated using 1,9-dimethylmethylene blue (DMMB) method. Forty microliters of papain digestive solution of each construct was added to 250 *μ*L of DMMB dye (pH 3.0) in a 96-well microtiter plate. After mixing, the absorbance of the solutions was measured at 595 nm and was compared with the linear standard curve obtained from the known concentration of 6-chondroitin sulfate. To determine the cell amount of each construct, DNA content was measured. 100 *μ*L of digestive solution was mixed with 1 mL of Hoechst 33258 dye/buffer, and 200 *μ*L of mixture was evaluated for the excitation at 365 nm and emission at 458 nm by the microplate absorbance reader. A standard curve was established from the known concentration of calf thymus DNA.

To detect the difference in chondrogenic gene expression between the two constructs, the ECM-related gene expression was evaluated with real-time PCR. Snap frozen constructs (*n* = 5 of each group) were pulverized in liquid nitrogen and total RNA was extracted using RNAiso Plus (TAKARA, D9108A). After elimination of genomic DNA contamination, 450 ng of total RNA was reverse transcribed into cDNA using Oligo (dT)_15_ as a reverse primer (TAKARA, DRR037A). Equivalent amounts of cDNA were used for real-time PCR in a 20 *μ*L reaction mixture with 10 *μ*L of 2x SYBR Green PCR Mastermix and 1 *μ*L of specific primer pair. Reaction was run in triplicate with 40 cycles of amplification on an ABI Prism 7500 real-time PCR (Applied Biosystems, USA). The sequences of primers were shown in Table 1. The expression levels of target genes were normalized by the expression of GAPDH gene measured in parallel samples. Relative transcript levels were calculated as *χ* = 2^−ΔΔCt^, in which ΔΔCt = Δ*E* − Δ*C*, Δ*E* = Ct_exp⁡_ − Ct_GAPDH_, and Δ*C* = Ct_ct1_ − Ct_GAPDH_.

### 2.6. *In Vivo* Animal Model to Evaluate the Repairing Ability

**Figure 1:**
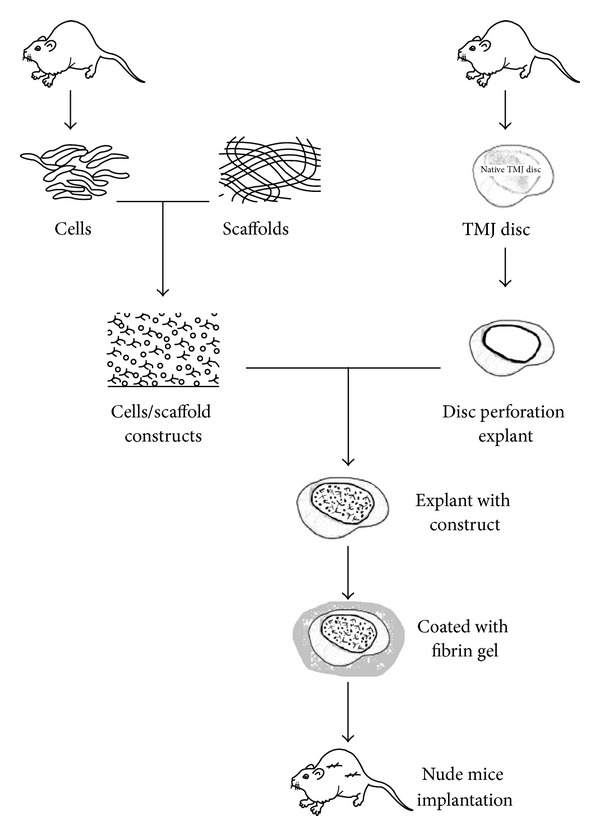
The experiment design and animal models for repairing TMJ disc perforation.

To evaluate the repairing ability of cell/scaffold constructs in defect of fibrocartilage, an animal model of TMJ disc perforation was designed (Figure 1).

**Figure 2:**
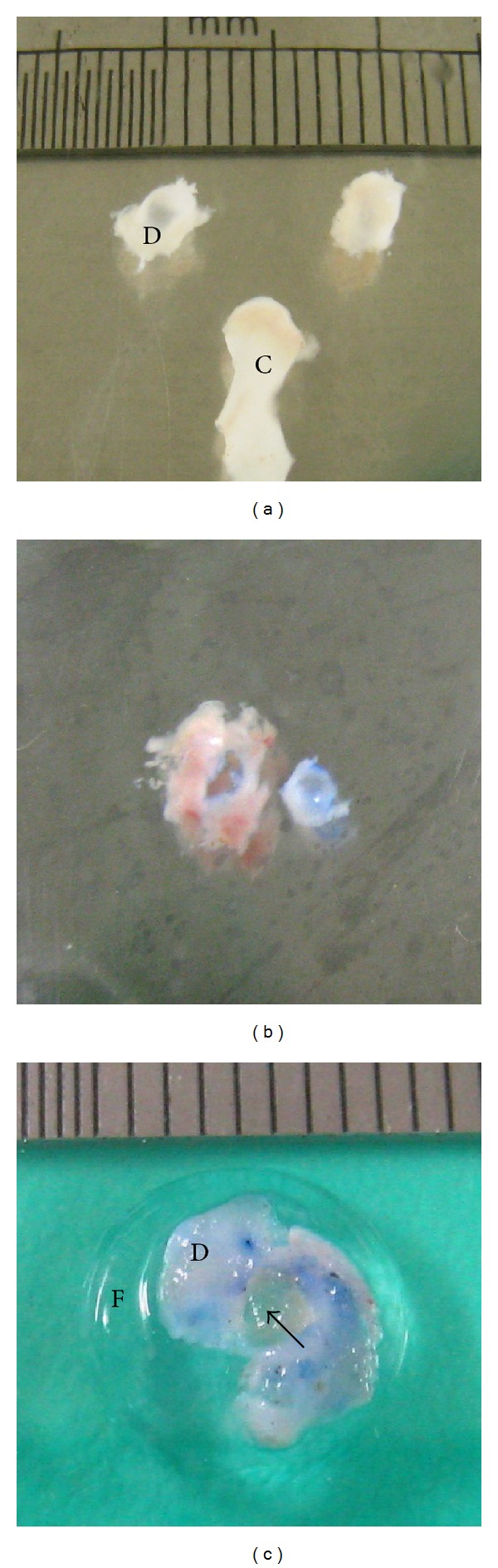
(a) Rat TMJ discs were obtained from mandibular condyle (C: condyle and D: TMJ disc); (b) TMJ disc perforation of 2 mm in diameter was surgically made by a puncher; (c) scaffolds with or without cells (arrow) were inserted into the perforation of disc and then coated the explants with pure fibrin before subcutaneous implantation (D: TMJ disc and F: fibrin).

Thirty rat TMJ discs were dissected and separated from mandibular condyle aseptically (Figure 2(a)). After washing in PBS, surrounding synovial tissue of the TMJ disc was removed under stereo microscope. A unified perforation of the TMJ discs was made by punching the disc body with a 2 mm diameter puncher (Figure 2(b)).

Chondrogenic induced constructs and cell-free constructs were punched into same size as disc perforation and were implanted into the site of TMJ disc perforation. Before subcutaneous transplantation, all the disc explants were coated with 2 mm thick fibrin gel to prevent exogenous host cells migration into the constructs (Figure 2(c)). Five groups of TMJ disc explants were set in Table 2.

The fibrin-coated TMJ discs explants were implanted subcutaneously in 2-week-old nude mice (Hubei Medical Laboratory Animal Center). After 4 weeks, mice were euthanize and explants were fixed for histological analysis (*n* = 5 of each group).

For histological analysis, samples were fixed overnight at 4°C in 4% paraformaldehyde in PBS. Samples were embedded in paraffin and sectioned to 5 *μ*m thickness. Consecutive sections were stained with HE and Safranin O/Fast Green for glycosaminoglycans. For immunohistological evaluations, collagen type I and type II were detected. In brief, sections were incubated with primary antibody against collagen type I and type II overnight at 4°C, followed by the secondary antibody of biotinylated goat anti-rabbit IgG, and detected by using ABC reagent with 3,3′-diaminobenzidine as a substrate. Negative control staining against each primary antibody was performed by replacing the primary antibody with distilled water to test the workability of the antibody. All the sections were counterstained with hematoxylin and observed by a light microscope (Leica, Wetzlar, Germany).

### 2.7. Statistical Analysis

Cell seeding efficiency, proliferation ability, GAG content, GAG/DNA ratio, and mRNA expression levels were compared between the scaffolds with or without fibrin gel incorporation, with a risk factor of less than 0.05 considered statistically significant. All data were expressed as mean ± standard deviation. Statistical differences were evaluated between the two constructs with Student's* t*-test and two-factor ANOVA using GraphPad Prism 4 statistical software (San Diego, USA).

## 3. Results 

### 3.1. Morphological Features of Fibrin/Chitosan Scaffold

**Figure 3:**
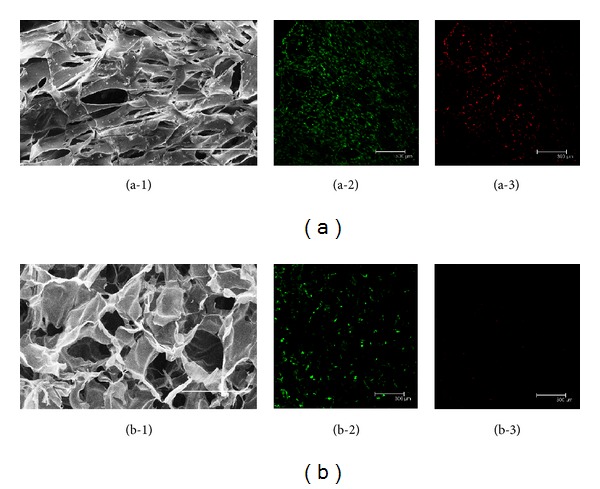
Morphologic features of two scaffolds. SEM results showed more cavities among pure chitosan scaffold (b-1), while the cavities were filled with fibrin in fibrin/chitosan scaffold (a-1). CLSM results showed that more vital cells (green) existed in fibrin/chitosan scaffold (a-2) than in chitosan scaffold (b-2), although the number of dead cells (red) was relatively higher in hybrid scaffold ((a-3) and (b-3)). Scale bar is equal to 300 *μ*m.

The pure chitosan scaffold demonstrated a macroporous structure fulfilled with cavity (Figure 3(b-1)). When combining chitosan with fibrin, the cavity of chitosan was extensively filled with fibrin (Figure 3(a-1)).

After 7 days of three-dimensional culture, constructs were stained with FDA/PI to assess cell viability and distribution 100 *μ*m below the surface. More vital cells were homogeneously distributed among fibrin/chitosan scaffolds (Figure 3(a-2)). Although many dead cells (red) were evident in fibrin incorporated scaffold (Figure 3(a-3)), the amount of green vital cells were higher than chitosan scaffold (Figure 3(b-2)).

### 3.2. Biocompatible and Biochemical Researches of TMJ-SDSCs Seeded into Two Scaffolds

**Figure 4:**
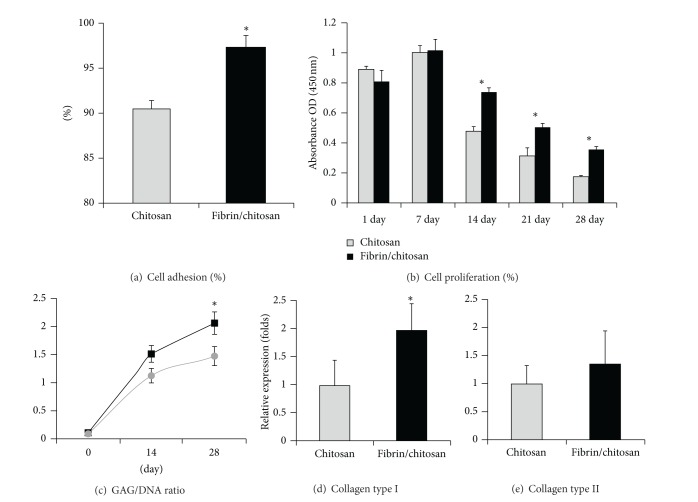
Biocompatible and biochemical results of TMJ-SDSCs seeded in two scaffolds. Columns and error bars represent means and SD. Cell adhesion (a), GAG/DNA ratio (c), and relative expression of Col I (d) were significantly improved in fibrin/chitosan scaffold. Asterisks (∗) indicate significant difference from control (*P* < 0.05), based on the* post hoc* analysis comparing each individual group. Cell proliferation results (b) demonstrated significant difference (*P* < 0.05) in the two-factor ANOVA (fibrin and time duration).

Comparing with the traditional seeding technique, the incorporation of fibrin gel may significantly improve cell seeding efficiency. In fibrin/chitosan constructs, more TMJ-SDSCs (97.28 ± 0.935%) were eventually seeded into the scaffold, while significantly less cells were retained in pure chitosan scaffold (90.46 ± 1.366%) (Figure 4(a)).

In cell proliferation assay, TMJ-SDSCs proliferated in both of the two scaffolds during the first week. From day 7 to day 28, the amount of live cells decreased gradually. Statistic differences were observed at days 14, 21, and 28, indicating a better biocompatibility of the fibrin/chitosan scaffold (Figure 4(b)).

Biochemical quantification of the total DNA amount and sGAG accumulation was performed during the 28 days of chondrogenic induction. In fibrin/chitosan constructs, a significantly higher sGAG/DNA ratio was achieved at 28 days, indicating a stronger sGAG synthetic ability (about 1.4-fold to control) of the cells (Figure 4(c)).

The relative mRNA levels of chondrogenic markers including collagen type I and type II were quantified using real-time RT-PCR. The expression of collagen type I mRNA in fibrin/chitosan scaffold was significantly higher (about 1.97-fold to control) than in pure chitosan scaffold (Figure 4(d)). However, no significant difference of collagen type II mRNA was observed among the two groups (Figure 4(e)).

### 3.3. *In Vivo* Subcutaneous Implantation of TMJ Disc Explants

**Figure 5:**
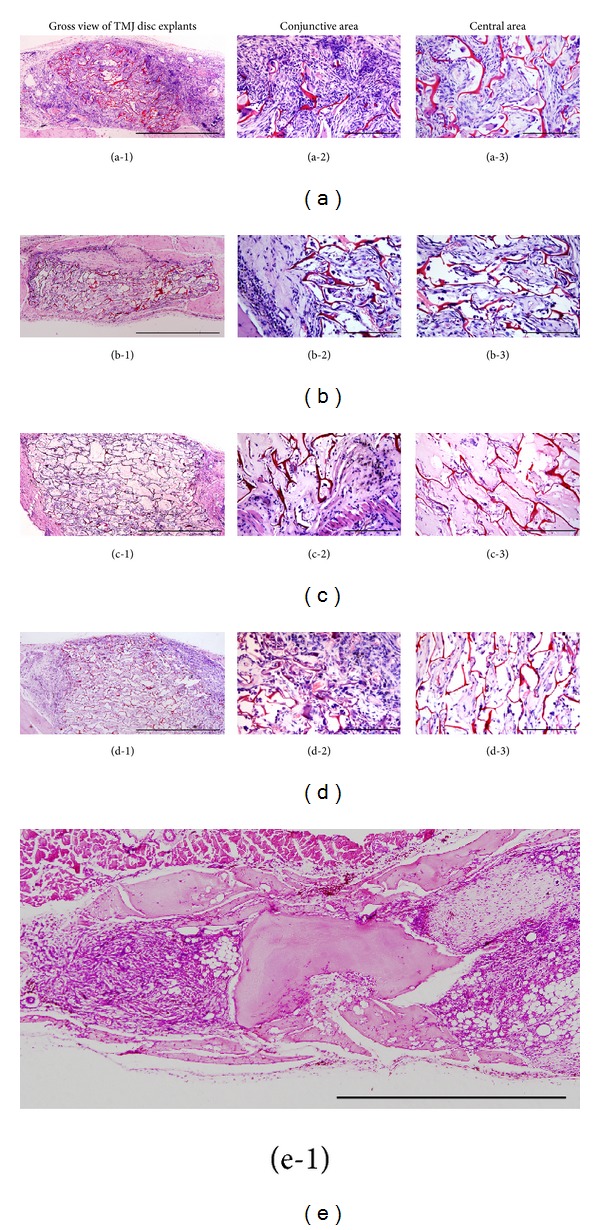
HE staining of TMJ disc explants. (a-1)–(e-1) are gross views of TMJ explants. Scale bar is equal to 1000 *μ*m; (a-2)–(d-2) are magnifications of the conjunction between constructs and native disc tissue; (a-3)–(d-3) are magnifications of the central part of scaffold; scale bar is equal to 100 *μ*m.

After 4 weeks of subcutaneous implantation, all samples were histologically analyzed. Gross view of HE staining demonstrated more matrix depositions with fewer cavities in fibrin/chitosan scaffold (Figure 5(a-1)) than fibrin-free constructs (Figure 5(b-1), Group B). Few host cells were observed in cell-free group with no sign of repair (Figures 5(c-1), 5(d-1), and 5(e-1)).

The conjunction between constructs and native disc tissue (Figures 5(a-2), 5(b-2), 5(c-2), and 5(d-2)) and central part of constructs (Figures 5(a-3), 5(b-3), 5(c-3), and 5(d-3)) was magnified to evaluate the repair capacity. In Group A (Figures 5(a-2) and 5(a-3)), high density of cells and dense staining of ECM accumulation were observed compared to Group B (Figures 5(b-2) and 5(b-3)). No sign of repair was found in cell-free groups (Figures 5(c), 5(d), and 5(e)). In the pure chitosan scaffold (Figure 5(d)), few host oriented cells aggregated to the framework of the chitosan scaffold. In fibrin/chitosan scaffold, host cells were distributed more separately along with the fibrin (Figure 5(c)). In the fibrin group, the perforation was still filled with fibrin without any sign of disc repair (Figure 5(e)).

**Figure 6:**
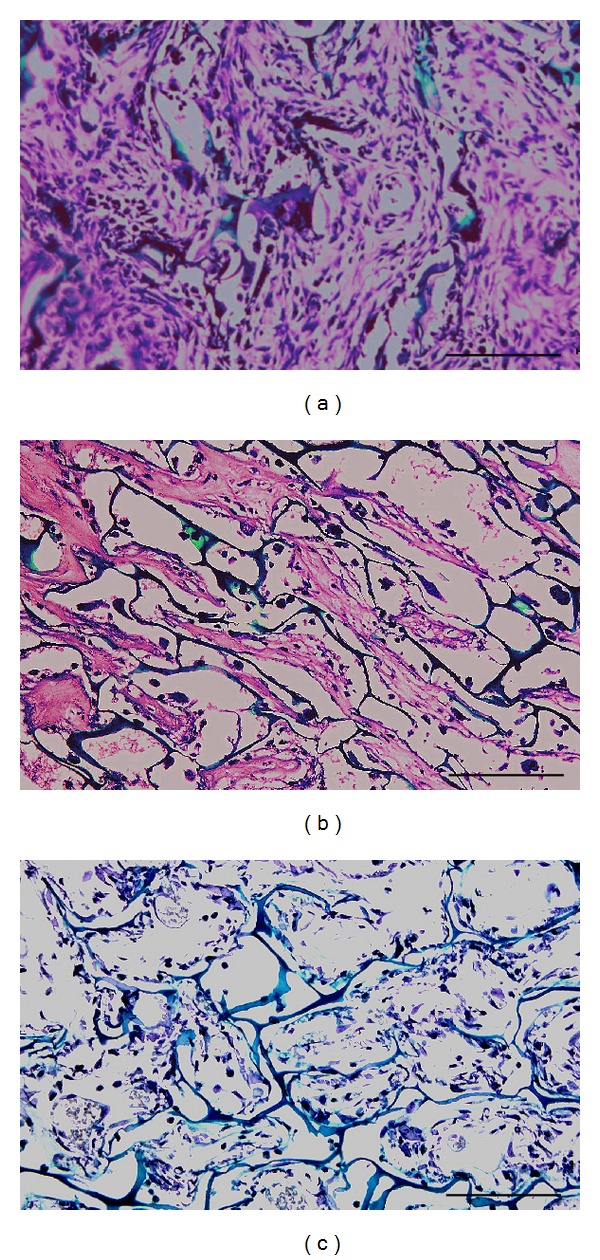
Safranin O/Fast Green staining of each group. Synthesized sGAG were stained red in Groups A and B. Chitosan scaffold was stained green and cell nuclei of cells were stained black. No sign of red staining in Group C indicated that fibrin gel was unable to be stained with safranin O. Scale bar is equal to 100 *μ*m.

Safranin O/Fast Green staining showed more sGAG accumulation in fibrin-added Group A constructs (Figure 6(a)) than fibrin-free Group B constructs (Figure 6(b)). In cell-free control group, no sGAG deposition was found (Figure 6(c)).

**Figure 7:**
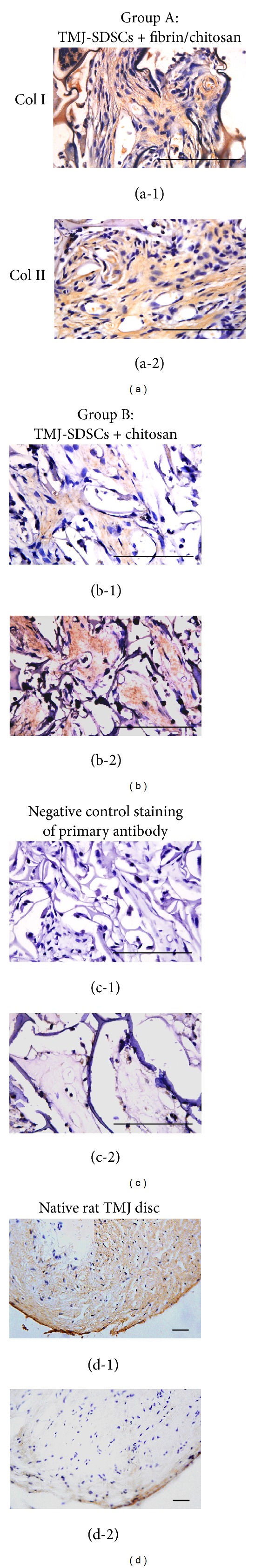
IHC staining of collagen type I ((a-1)–(d-1)) and type II ((a-2)–(d-2)). (a) Group A explants; (b) Group B explants; (c) negative control of primary antibody; (d) native rat TMJ disc. These results confirmed that TMJ-SDSCs may synthesis both collagen types I and II after chondrogenic induction. (d-1) and (d-2) confirmed that collagen type I was the major component of native TMJ disc. Scale bar is equal to 100 *μ*m.

In accordance with mRNA expression results, staining of collagen type I and collagen type II was more intensive in fibrin-added constructs (Figures 7(a-1), 7(a-2)) than in fibrin-free constructs (Figures 7(b-1), 7(b-2)) at 4 weeks. Negative control staining of each primary antibody in Group A specimens was shown in Figures 7(c-1) and 7(d-1), respectively.  Figure 7(d) confirmed that strong expression of collagen type I rather than collagen type II was demonstrated in native TMJ disc. However, when compared with native TMJ disc (Figure 7(d-2)), over expression of collagen type II was noted in SDSCs seeded groups (Figures 7(a-2) and 7(b-2)).

## 4. Discussions

Although many researches focused on cartilage regeneration, only a handful of studies were on TMJ disc engineering. In the field of cartilage engineering, cell source and matrix scaffold were two major factors. This research is the first* in vivo* study using TMJ-SDSCs in TMJ disc engineering and confirms that SDSCs obtained from TMJ synovium may undergo chondrogenic differentiation in a general accepted manner. The addition of fibrin gel enhanced the ability of cartilage ECM production of the TMJ-SDSCs.

In previous study of TMJ disc engineering, the cells were harvested from the disc [3, 5, 6, 14] or from hyaline cartilage [15, 16]. However, the drawbacks of using fibrochondrocyte/chondrocyte including difficult cell harvesting, injury to donor site, and cell dedifferentiation among* in vitro* expansion hindered their utility in cartilage engineering [7, 17, 18].

Bone marrow mesenchymal stem cells (BMSCs) were superior in cell proliferation and chondrogenic differentiation ability. However, researchers found that BMSCs can express collagen type X after chondrogenic induction and tend to undergo endochondral ossification after subcutaneous implantation [19]. MSCs derived from the joint, including adipose-derived stem cells (ADSCs) and SDSCs are promising alternative cell sources that may overcome the intrinsic drawbacks of using chondrocytes [20, 21]. SDSCs have been shown to be prior to ADSCs in cell expansion ability and chondrogenesis potential [20]. In clinical researches, synovial chondromatosis could be found within human TMJ synovial tissues, which illustrates the potential of some subpopulations of TMJ synovial cells to transform into chondrocytes and form cartilage-like tissue [22]. Miyamoto et al. found that SDSCs could be recruited* in vivo* to repair adjacent partial-thickness cartilage defects [23]. These evidences imply the possibility of using TMJ-SDSCs in TMJ disc tissue regeneration.

The synovial membrane consists of type-A macrophage-like cells and type-B SDSCs [24]. SDSCs can be generally purified by continuous passage as the poor proliferation ability of type-A cells. However, SDSCs obtained with the conventional method may not be completely pure. Bilgen et al. [25] purified the SDSCs using CD14-negative isolation method and found that purified SDSCs demonstrated higher expression of collagen type II and aggrecan as well as lower expression of collagen type I under the same condition. Their results might be in favour of hyaline cartilage regeneration but might not refer to fibrocartilaginous repair especially in TMJ disc, which is predominately consisted as collagen type I with collagen type II in trace amount [26, 27]. So in this study, TMJ-SDSCs were isolated with the conventional methods and the results show that both collagen type I and cartilage-related ECM including collagen type II and sGAG were synthesized after chondrogenic induction.

Biomaterials used in previous cartilage engineering researches can be divided as hydrogels, fibrous meshed, and sponges with respect to the scaffold structure. Several synthetic materials had been used in TMJ disc engineering, including polylactide (PLA), polyglycolic acid (PGA), and their copolymers (PLGA) [28]. Natural chitosan is polysaccharide material which has been used extensively in the field of cartilage regeneration for its good biocompatibility and similarity to GAG cartilage [29]. Porous chitosan scaffold provides mechanical strength and shape-persistency during both* in vitro* and* in vivo* cultures, which has been confirmed in our researches [12]. However, cell seeding onto this kind of scaffold is unlikely to be homogeneous. Most cells tend to adhere only on the scaffold surface. So improving cell seeding and distribution is essential for the limited amount of joint derived cells.

Hydrogels have great scaffolding potential due to their high biocompatibility, efficient transport of nutrients and waste, ability to uniformly encapsulate cells, and ability to be made into any shape [30]. Besides the clinical application as haemostatic sealant [31], fibrin gel has been widely used in a variety of tissue engineering including cardiovascular [32], liver [33], skin [34], bone [35], and cartilage tissues [36, 37]. It was reported that fibrin gel may benefit chondrocytes for maintaining stable phenotype and synthesizing cartilage ECM in nude mice [38]. Izuta et al. [39] found that fibrin combined with MSCs may promote avascular zone of meniscal healing after 8 weeks of implantation.

The major disadvantages of using fibrin gel as scaffold include the shrinkage of the volume during gel formation, poor mechanical stiffness, and rapid degradation [32]. In this study, fibrin gel was incorporated with porous chitosan scaffold. We hypothesized that this hybrid scaffold may promote cell differentiation and ECM synthesis and may be fixed in the site of disc defect by the adhesive feature of fibrin gel. After cell seeding into this fibrin/chitosan scaffold, a higher seeding efficiency and more homogeneous cell distribution was observed compared with fibrin-free scaffolds. These results were in accordance with Swartz et al.'s study [40]. From day 1 to day 7, TMJ-SDSCs seemed to proliferate quicker in fibrin-added scaffolds than in fibrin-free scaffolds, although no statistic difference was observed. Cell numbers dramatically decreased in both groups from day 7 to day 28. Statistic differences were observed at these time points, indicating a better maintenance of cell viability presented in fibrin-added scaffolds.

In the present research, TGF-*β*3 was the only growth factor used in chondrogenic induction. Lee et al. [41] compared the use of TGF-*β* alone with a combination of TGF-*β* and BMP in chondrogenic induction of calf SDSCs and found that the gene expression of collagen type II, aggrecan, and SOX9 increased along with the addition of BMP-7; however, the expression of collagen type I decreased. In fibrocartilage, collagen type I was predominant, while collagen type II was found in small amount. So in this study, only TGF-*β*3 was used. After chondrogenic induction, sGAG were synthesized by the TMJ-SDSCs and deposited in scaffolds. The sGAG/DNA ratio indicates the synthetic ability of cells. In fibrin-added groups, sGAG/DNA ratio was higher than fibrin-free groups at day 14 and day 28.

In animal model study, TMJ-SDSCs seeded fibrin/chitosan constructs showed better repair than fibrin-free constructs after 4 weeks of subcutaneous implantation. Most of the cells in Group A were rounded rather than spindle-shaped in fibrin-free group. According to previous researches on the characterization of animal TMJ disc, approximately 2/3 of the cells in the disc are fibroblast-like cells and the rest are chondrocyte-like cells [42]. Histological results including the staining of Col I, Col II, and sGAG were superior in fibrin-added constructs, which was in accordance with the cartilage-related mRNA expression.

In summary, fibrin gel improved the synthesis of fibrocartilage ECM by TMJ-SDSCs. This pilot study demonstrated that the regenerative ability of TMJ-SDSCs seeded fibrin/chitosan constructs could be applied for repairing TMJ disc perforation.

## Figures and Tables

**Table 1:** The primer sequences of ECM related genes.

Genes	Primer sequence (F, R, 5′→3′)	Product length (bp)	GenBank accession number
Col 1A1	CCTACAGCACGCTTGTGGATG	195	NM_053304.1
	AGATTGGGATGGAGGGAGTTTAC		
Col 2A1	GACTTTCCTCCGTCTACTGTCC	171	NM_012929.1
	GTGTACGTGAACCTGCTGTTG		
GAPDH	GGCACAGTCAAGGCTGAGAATG	143	NM_017008.4
	ATGGTGGTGAAGACGCCAGTA		

**Table 2:** 

Group A	TMJ disc within chondrogenic induced cell/fibrin/chitosan scaffold
Group B	TMJ disc within chondrogenic induced cell/chitosan scaffold
Group C	TMJ disc within cell-free fibrin/chitosan scaffolds
Group D	TMJ disc within cell-free chitosan scaffold
Group E	TMJ disc within cell-free fibrin scaffold
